# Exploring Microemulsion Systems for the Incorporation of Glucocorticoids into Bacterial Cellulose: A Novel Approach for Anti-Inflammatory Wound Dressings

**DOI:** 10.3390/pharmaceutics16040504

**Published:** 2024-04-07

**Authors:** Paul Zahel, Vera Bruggink, Juliana Hülsmann, Frank Steiniger, Robert K. Hofstetter, Thorsten Heinzel, Uwe Beekmann, Oliver Werz, Dana Kralisch

**Affiliations:** 1 JeNaCell GmbH—An Evonik Company, 07745 Jena, Germany; paul.zahel@evonik.com (P.Z.); vera.bruggink@uni-jena.de (V.B.); uwe.beekmann@evonik.com (U.B.); 2 Department of Pharmaceutical/Medicinal Chemistry, Institute of Pharmacy, Friedrich Schiller University, 07743 Jena, Germany; robert.klaus.hofstetter@uni-jena.de (R.K.H.); oliver.werz@uni-jena.de (O.W.); 3 Institute of Biochemistry and Biophysics, Center for Molecular Biomedicine, Friedrich Schiller University, 07745 Jena, Germany; juliana.huelsmann@uni-jena.de (J.H.); t.heinzel@uni.jena.de (T.H.); 4 Electron Microscopy Center, University Hospital Jena, Friedrich Schiller University, 07743 Jena, Germany; frank.steiniger@med.uni-jena.de; 5 Evonik Operations GmbH, 45128 Essen, Germany

**Keywords:** bacterial cellulose, glucocorticoids, anti-inflammatory, pyoderma gangraenosum, microemulsion, wound care, drug delivery, wound dressing, hydrocortisone, dexamethasone

## Abstract

The effective pharmacological treatment of inflamed wounds such as pyoderma gangraenosum remains challenging, as the systemic application of suitable drugs such as glucocorticoids is compromised by severe side effects and the inherent difficulties of wounds as drug targets. Furthermore, conventional semi-solid formulations are not suitable for direct application to open wounds. Thus, the treatment of inflamed wounds could considerably benefit from the development of active wound dressings for the topical administration of anti-inflammatory drugs. Although bacterial cellulose appears to be an ideal candidate for this purpose due to its known suitability for advanced wound care and as a drug delivery system, the incorporation of poorly water-soluble compounds into the hydrophilic material still poses a problem. The use of microemulsions could solve that open issue. The present study therefore explores their use as a novel approach to incorporate poorly water-soluble glucocorticoids into bacterial cellulose. Five microemulsion formulations were loaded with hydrocortisone or dexamethasone and characterized in detail, demonstrating their regular microstructure, biocompatibility and shelf-life stability. Bacterial cellulose was successfully loaded with the formulations as confirmed by transmission electron microscopy and surprisingly showed homogenous incorporation, even of w/o type microemulsions. High and controllable drug permeation through Strat-M^®^ membranes was observed, and the anti-inflammatory activity for permeated glucocorticoids was confirmed in vitro. This study presents a novel approach for the development of anti-inflammatory wound dressings using bacterial cellulose in combination with microemulsions.

## 1. Introduction

Successful treatment and management of chronic, non-healing wounds continue to be a major challenge for healthcare providers and play a critical role in maintaining patients’ quality of life. In particular, the treatment of inflammatory dermatoses such as pyoderma gangrenosum relies not only on the use of modern and adaptive wound management but also on effective pharmacotherapy involving the use of glucocorticoids [1].

Glucocorticoids, such as hydrocortisone (HC) and dexamethasone (DEX), are synthetic steroid hormone derivatives that are extensively used for their anti-inflammatory and immunosuppressive effects [2]. The mechanisms underlying these effects involve both genomic and non-genomic pathways, although the molecular processes of all these pathways have not yet been conclusively understood [3].

The genomic mechanism is mediated via the cytosolic glucocorticoid receptor (GR) and mainly affects the upregulation or downregulation of gene transcription. Upon cellular entry of the lipophilic glucocorticoids through passive diffusion, the molecules bind to the GR within the cytoplasm. The formed glucocorticoid receptor complex (GRC) translocates to the nucleus and binds directly to glucocorticoid response elements (GREs) in the promoter regions of glucocorticoid-dependent target genes. The binding of the GRC to positive GREs activates the transcription of anti-inflammatory proteins such as interleukin (IL)-10, annexin A1 and the inhibitor of nuclear factor κB (NFκB), termed IκB. This process, which involves the stimulation of gene transcription via direct DNA binding, is associated with numerous adverse glucocorticoid effects and is called transactivation [3,4,5].

The second mechanism of genomic glucocorticoid action, termed transrepression, involves the direct inhibition of the transcription of pro-inflammatory genes, e.g., pro-opiomelanocortin, by the binding of the GRC to negative GREs [6]. Moreover, the GRC can inhibit the effects of several transcription factors, such as NFκB and activating protein-1 (AP-1) through direct and indirect interactions, preventing their DNA binding and therefore suppressing the synthesis of pro-inflammatory cytokines, e.g., IL-1, IL-2, IL-6, tumor necrosis factor α (TNFα) and interferon γ [3,7,8]. The transrepression is currently believed to be primarily responsible for the desired clinical anti-inflammatory and immunosuppressive effects with a particular focus on the effect of NFκB and AP-1 inhibition [3,5]. In addition to these genomic mechanisms, it is important to mention that some of the anti-inflammatory and immunosuppressive effects of glucocorticoids that can be observed shortly after the intravenous administration of high glucocorticoid doses occur too fast to be attributed to the genomic modes of action. They are believed to be mediated through nonspecific interactions of glucocorticoids with cellular membranes, specific interactions with membrane-bound GRs and non-genomic effects mediated by the cytosolic GR [3,9,10,11,12]. Although glucocorticoid use can be associated with impaired wound healing, this has only been confirmed for high-dose systemic application [13]. Since the systemic application of glucocorticoids is also connected with several severe side effects [14], the topical administration in contrast can be a promising option and is even recommended by medical guidelines in combination with systemic use [15,16]. Unfortunately, many challenges remain in the context of successfully delivering active pharmaceutical ingredients (APIs) to wounds. The use of conventional, non-sustained dosage forms that need daily application is not reconcilable with a desired reduction in dressing change frequency to increase patient compliance [17]. Moreover, systemic drug delivery to wounds is particularly challenging due to obstacles such as ineffective vascularization of the wound bed and thus impaired transport of active agents through the blood flow [18,19]. These problems propose the need for a suitable wound dressing that is mechanically stable, capable of delivering active ingredients while maintaining wound healing promoting a moist wound environment, sufficient gas exchange and proper mechanical barrier functions. It shall maintain these functions over several days until dressing change.

Bacterial cellulose (BC), also known as biosynthetic cellulose, is a unique biopolymer produced by several bacteria species such as *Komagataeibacter xylinus* from glucose monomers and other carbon sources. The material consists of a three-dimensional network of interconnected cellulose fibers with an exceptionally high water content, characterizing it as a form of stable hydrogel [20,21].

Its application as a wound dressing material was already described in the 1980s and has been the subject of intense research ever since [22,23]. In this respect, not only the high biocompatibility, mechanical stability and gas permeability are of particular advantage [23,24]. Clinical data have shown that wound dressings based on BC are able to relieve pain, promote re-epithelization and absorb wound exudate while providing a moist wound milieu [25]. In particular, the removal of plaque and fibrous tissue via autolytic debridement is significantly enhanced by the moisture donated from BC-based wound dressings and enables successful wound bed preparation [25,26,27]. Furthermore, the large surface area and water-holding capacity enable the uptake and release of active compounds, even with the possibility of controlling release kinetics [23,28]. Therefore, BC represents a promising candidate for the development of bioactive dressings to deliver anti-inflammatory agents, if the beneficial properties of the wound dressing itself can be maintained.

As the native BC material consists of more than 90% water, the incorporation of poorly water-soluble compounds such as glucocorticoids still remains challenging [29]. Previously, strategies to integrate lipophilic compounds focused on drug solubility improvement, the surface modification of BC fibers and the formulation of dispersed systems [30]. In the case of the latter, recent studies investigating nanoemulsions [29,31], liposomes [29] or polymeric microparticles [32], are particularly worthy of mention. However, these approaches have a number of limitations.

Improving API solubility via salt formation requires certain chemical conditions and high development effort and can significantly influence the physical and chemical properties of the API [30,33]. Surface modification of BC fibers and in situ microparticle formation require the use of organic reagents and solvents, the residues of which must be carefully removed before medical use and may delay the registration process [30,32,34,35]. Nanoemulsions are thermodynamically unstable, and phase separation due to coalescence, flocculation and Ostwald ripening can be an issue for shelf-life stability, which needs careful formulation design [36]. Besides that, most nanoemulsion preparation methods rely on emulsification techniques that are difficult to scale up or require expensive preparation equipment [36,37].

A novel approach, namely the use of ready-formulated microemulsions (MEs) for the incorporation of anti-inflammatory compounds such as glucocorticoids into BC, may overcome the limitations of these earlier concepts.

MEs are thermodynamically stable, optically isotropic and transparent/translucent mixtures of oil and water stabilized by at least one surfactant. They form spontaneously at specific compositions of the two phases, an amphiphilic surfactant and often one cosurfactant (usually short-chain alcohols) which interacts with the amphiphilic layer by increasing its curvature and fluidity, thus enabling extremely low interfacial tension [38,39,40,41]. The microstructure of MEs can be classified either as bicontinuous or dispersed, depending on the concentration and character of its constituents. While bicontinuous MEs are characterized by continuous domains of water and oil, dispersed MEs more closely resemble droplet structures which typically show sizes between 3 nm and 50 nm [38,41]. As a consequence of their unique properties, MEs feature a wide range of advantages for their use as a drug carrier, which include long-term storage stability, facile preparation and favorable solubilization capacity for hydrophilic and lipophilic drugs [41]. In addition, previous investigations have shown that MEs facilitate the penetration of drugs through the stratum corneum and accumulation within the skin [42]. There are numerous hypotheses for this effect, such as a high concentration gradient toward the skin as a consequence of high drug solubility, a penetration enhancement effect of amphiphilic molecules through the disruption of stratum corneum lipids or an increase in the partition coefficient of the drug between skin and formulation and the high internal mobility of the drug in the vehicle [43,44]. Based on these properties, MEs were chosen in this study as a vehicle to incorporate lipophilic, poorly water-soluble glucocorticoids into aqueous and hydrophilic BC to develop active, anti-inflammatory wound dressings for inflamed, chronic wounds such as pyoderma gangraenosum. To our best knowledge, this trial is one of the first studies to examine the loading of ready-formulated MEs into BC, as well as one of the first studies that developed a solution to incorporate glucocorticoids into the material. It is important to note that Marquele-Oliveira et al. reported the development of an innovative wound dressing based on BC by the incorporation of a self-microemulsifying formulation with a solvent exchange method to deliver propolis [45]. Whereas this approach focused on a naturally derived ingredient, Valo et al. investigated the incorporation of beclomethasone dipropionate nanoparticles into several nanofibillar cellulose aerogels, one of them obtained from BC, demonstrating a possibility of incorporating glucocorticoids into BC [46]. Furthermore, Rojewska et al. studied the properties of bioactive dressings based on carboxymethylated BC that were functionalized with dexamethasone phosphate-loaded submicroparticles via carbodiimide chemistry [47].

This study focuses on the development of active, anti-inflammatory wound dressings for the treatment of pyoderma gangraenosum by a novel approach to directly load ready-formulated MEs containing hydrocortisone (HC) or dexamethasone (DEX) into native BC dressings. The formulations were characterized in detail regarding microstructure, drug storage and sterilization stability and their biocompatibility via measuring of in vitro cytotoxicity. Pure and incorporated formulations were imaged by transmission electron microscopy in a freeze–fracture technique. Loading characteristics, in vitro permeation through Strat-M^®^ membranes and anti-inflammatory activity efficacy after release and permeation were evaluated to address particularly important aspects in the development of an active wound dressing.

## 2. Materials and Methods

### 2.1. Construction of Pseudo-Ternary Phase Diagram and ME Components

For ME formulation, component mixtures were chosen according to Fini et al. [48]: surfactant:cosurfactant mixture (Smix) consisted of Labrasol^®^ as main surfactant and Transcutol^®^ P as cosurfactant in the ratio of 5:1 (*w*/*w*), while oil phase (Omix) consisted of Plurol Oleique^®^ CC 497 and Labrafil^®^ M 1944 CS in 1.4:1 ratio (*w*/*w*) (all kindly gifted by Gattefossé, Lyon, France). To determine the ME region, a pseudo-ternary phase diagram was constructed using the water titration method [49]. Briefly, Smix and Omix were mixed in ratios of 1:19, 2:18, 3:17, 4:16, 5:15, 6:14, 7:13, 8:12, 9:11, 10:10, 11:9, 12:8, 13:7, 14:6, 15:5, 16:4, 17:3, 18:2 and 19:1 (*w*/*w*) until homogenous mixtures of 10 mL were obtained. Deionized water was added dropwise under stirring (Stirring Plate VMS-C7 Advanced, VWR International GmbH, Radnor, PA, USA). The total quantity of added deionized water was recorded at the transition point of transparency to turbidity, which represented the end point of the titration. The isotropic region presenting transparent, single-phase and low-viscous mixtures was plotted on the pseudo-ternary phase diagram [49,50].

### 2.2. Preparation of ME Formulations and Glucocorticoid Loading

For the formulation of MEs, five mixtures (ME-A, ME-B, ME-C, ME-D, ME-E) with varying ratios of deionized water, Smix and Omix were selected as listed in Table 1. The components were weighed in glass vials. They all spontaneously formed MEs after short periods of stirring. All formulations were stirred for 60 min at minimum to ensure complete homogenization.

For the preparation of HC-loaded MEs, micronized hydrocortisone powder (Euro OTC Pharma GmbH, Bönen, Germany) was added to the MEs at 0.5 wt% and dissolved under stirring. For DEX-loaded MEs, micronized dexamethasone powder (Euro OTC) was added to the MEs at 0.05 wt% and dissolved under stirring. The obtained samples were labeled and are referred to as follows:

- ME-A; ME-B; ME-C; ME-D; ME-E (blank MEs without API).
- ME-A-HC; ME-B-HC; ME-C-HC; ME-D-HC; ME-E-HC (HC-loaded MEs).
- ME-A-DEX; ME-B-DEX; ME-C-DEX; ME-D-DEX; ME-E-DEX (DEX-loaded MEs).

### 2.3. Microemulsion Characterization

#### 2.3.1. Rheology

To determine the viscosity, rheological studies of all five MEs were performed using a Brookfield DV-II + viscometer (AMETEK Brookfield, Middleboro, MA, USA)fitted with an Ultra Low Viscosity Adapter and a ULA 31 Y spindle. The sample temperature was set to 25 °C by a water bath. Viscosity [mPa·s], shear stress [Pa] and shear rate [1/s] measurements were performed at varying torques. Flow behavior was assessed by plotting shear stress vs. shear rate. The viscosity of each ME was determined as the mean value of viscosities at six suitable torques. All measurements were carried out in triplicate.

#### 2.3.2. Dynamic Light Scattering and Zeta Potential

Undiluted ME samples were characterized by laser light scattering and laser Doppler anemometry (Zetasizer Nano ZS, Malvern Panalytical Ltd., Worcestershire, UK). Hydrodynamic diameter measurements were carried out with a 4 mW He-Ne laser (633 nm) at a scattering angle of 173° at 25 °C in disposable minimal-volume cuvettes (Brand, Wertheim, Germany). Z-average was calculated as mean value of five measurements, and experiments were carried out in triplicate [49,51].

Zeta potential of all five MEs was determined at 25 °C in a Zetasizer DTS1070 Folded Capillary Cell (Malvern Panalytical Ltd.) using the same instrument. Results were calculated using Malvern Zetasizer Software 8.02 and are displayed as mean ± SD of 10 measurements. Experiments were performed in triplicate.

#### 2.3.3. Electrical Conductivity

To determine the conductivity of MEs and identify their microstructure, different ratios of Smix and Omix were prepared as described previously. For all mixtures, the conductivity was measured after addition of deionized water in steps of 5 wt% by using a Zetasizer Nano ZS and a DTS 1070 Folded capillary cell (Malvern Panalytical Ltd.). The conductivity of each sample was calculated as the mean of 10 measurements [51]. Linear fitting was performed using OriginPro 2023 (OriginLab, Northampton, MA, USA).

#### 2.3.4. pH, Refractive Index and Isotropy

For measuring the pH value of each ME, a calibrated pH meter (Mettler Toledo Five Easy, Greifensee, Switzerland) was used. Measurements were performed by direct immersion at 25 °C. All measurements were carried out in triplicate.

Refractive indices of all five MEs were determined at 25 °C using an Abbe refractometer (Krüss Optronic™ AR4, Hamburg, Germany).

The isotropy of all five ME formulations was evaluated using a polarizing light microscope at 100× magnification (ZETOPAN, C. Reichert Optische Werke AG, Vienna, Austria).

#### 2.3.5. Thermodynamic Stability

For evaluation of the thermodynamic stability of MEs, the samples were subjected to centrifugation and freeze–thaw test. Briefly, the samples were centrifuged at 4000 rpm for 30 min (Sigma 2-16P benchtop centrifuge, Sigma, Osterode am Harz, Germany). Furthermore, the samples were stored for 24 h at freezing (−20 °C) and room temperature (25 °C) with two replications of this cycle. Subsequently, the samples were checked for phase separation and transparency (*n* = 3) [52,53].

#### 2.3.6. Freeze–Fracture Transmission Electron Microscopy (FF-TEM)

For TEM imaging of all five MEs, aliquots of the samples were enclosed between two 0.1 mm thick copper profiles, as used for the sandwich double-replica technique. The sandwiches were physically fixed by rapid plunge freezing in a liquid ethane/propane (1:1) mixture cooled by liquid nitrogen. Freeze-fracturing was performed in a BAF400T freeze–fracture unit (BAL TEC, Balzers, Liechtenstein) at −140 °C using a double-replica stage. The fractured samples were shadowed without etching with a 2 nm thick platinum/carbon (Pt/C) layer at an angle of 35°, followed by a 15 nm thick carbon layer evaporated at an angle of 90°. Then, the samples were removed from the freeze–fracture unit and thawed. The freeze–fracture replicas were floated on water and picked onto copper EM grids (400 mesh). The freeze–fracture replicas were examined in a digitally upgraded Point Electronic Zeiss EM900 electron microscope (Point Electronic, Halle, Germany; Zeiss, Oberkochen, Germany) operated at 80 kV. Digital images were taken with a wide-angle dual-speed 2K CCD camera controlled by a Sharp/Eye base controller and operated by the Image SP software version 1.2.13.29 (camera and software TRS, Moorenweis, Germany).

#### 2.3.7. In Vitro Cytotoxicity

Human keratinocytes (HaCaT) [54] (CLS Cell Lines Service, Eppelheim, Germany, 300493) were grown in StableCell^TM^ Dulbecco’s Modified Eagle Medium (DMEM)-High glucose (Sigma-Aldrich, St. Louis, MO, USA) supplemented with 10% fetal calf serum (FCS) and 1% Penicillin-Streptomycin (P/S) (both Gibco, Thermofisher^TM^, Waltham, MA, USA). The cells were cultured at 37 °C, 5% CO_2_ and 95% RH.

For cytotoxicity evaluation, an MTT assay of blank and HC- and DEX-loaded MEs was performed. Therefore, about 15,000 HaCaT cells per well were seeded into a 96-well plate (Greiner Bio-One, Frickenhausen, Germany) and incubated for 24 h. Briefly, a dilution series of each sample was prepared to range from 960 to 1.875 µg/mL. The dilution was performed with DMEM + 10% FCS + 1% P/S. Afterward, 100 µL of each concentration was added per well. The plates were incubated for further 24 h. Next, the samples were aspirated and replaced through a 500 µg/mL solution of MTT (3-(4,5-dimethylthiazol-2-yl)-2,5-diphenyltetrazolium bromide, Alfa Aesar, Haverhill, MA, USA), which was incubated for 4 h. Subsequently the MTT was removed, and isopropyl alcohol (Carl Roth, Karlsruhe, Germany) was added to solve the violet dye. The plate was shaken for 10 min, followed by absorbance measurement at 570 nm using a FLUOstar OPTIMA Platereader (BMG Labtech, Ortenberg, Germany). Untreated cells served as 100% control, and a thiomersal 0.02% solution served as positive control (Caelo, Hilden, Germany). Cell viability was assessed by subtraction of the isopropyl alcohol blank value and calculation of the percentage of viable cells compared to the untreated cells. According to DIN EN ISO 10993-5 (10993-5, 2009) [55], values below 70% were identified as toxic. Samples were prepared in quadruplicates, and the test was performed thrice. Data are displayed as percentages (mean).

### 2.4. Stability Testing

#### 2.4.1. Storage Stability

For chemical stability testing, HC and DEX MEs were stored at 4 ± 2 °C in a refrigerator, at room temperature (21 ± 2 °C) and in accelerated conditions at 40 ± 2 °C and 75 ± 5% RH (ICH260 climate chamber, Memmert, Schwabach, Germany) in tightly sealed glass vials, protected from light. To investigate the effect of trace metals, metal complexing agent ethylene diamine tetraacetic acid disodium salt dihydrate (EDTA, Carl Roth) was added to aliquots of the MEs at 0.1 wt% and stored in the same way. Samples were withdrawn after 30 and 90 days of storage, and drug content of the samples was quantified immediately after sampling via Ultra High Performance Liquid Chromatography (UHPLC), after appropriate dilution with methanol (Carl Roth). The results were expressed as mean ± SD of percentual drug content in relation to control (0 days), experiments were performed in triplicate.

#### 2.4.2. Sterilization Stability

Stability of HC and DEX in ME formulations during different sterilization procedures was analyzed by UHPLC quantification of drug content of autoclaved and e-beam sterilized samples, in comparison to unprocessed control samples. To test autoclave resistance, 5 mL of all five MEs, loaded with either HC or DEX was transferred to amber glass vials and sterilized by autoclaving (121 °C, 2 bar, 15 min) in a table-top autoclave (D-23, Systec GmbH & Co. KG, Linden, Germany). To investigate the influence of e-beam irradiation on drug content, 5 mL aliquots of each sample were transferred to commercially available aluminum pouches, and e-beam sterilization was performed according to company internal procedures (JeNaCell GmbH, Jena, Germany). Drug content of the samples was quantified immediately after the sterilization procedures by UHPLC, after dilution with methanol (Carl Roth). The experiments were performed in triplicate, and results show mean percentual drug content in relation to control ± SD.

### 2.5. Preparation and Microemulsion Loading of BC

BC was synthesized under static cultivation of bacteria strain *Komagataeibacter xylinus* DSM 14666, deposited at the German Collection of Microorganism and Cell Cultures (DSMZ, Braunschweig, Germany) in a 1 m^2^ pilot plant as described previously [56,57]. For all experiments, circular patches of 15 mm diameter were used.

Microemulsion-loaded bacterial cellulose (BC-ME) was obtained by absorption loading technique as described previously [34]. Briefly, aliquots of 10 g of all five MEs were transferred separately to glass vials. For each individual sample, a circular BC patch of 1.0 ± 0.2 g was dehydrated to a weight of 0.20 ± 0.01 g with manual pressure and submersed in the ME aliquots. The absorption loading was performed for 72 h on a magnetic stirrer at 1000 rpm, and the patches were evaluated macroscopically and by determination of the patch weight. BC-ME samples containing HC and DEX were prepared in the same way using HC- and DEX-loaded ME, as described before (Section 2.2). The obtained BC patches are referred to as follows:

- BC-ME-A; BC-ME-B; BC-ME-C; BC-ME-D; BC-ME-E (ME-loaded BC without API).
- BC-ME-A-HC; BC-ME-B-HC; BC-ME-C-HC; BC-ME-D-HC; BC-ME-E-HC (ME-loaded BC containing HC).
- BC-ME-A-DEX; BC-ME-B-DEX; BC-ME-C-DEX; BC-ME-D-DEX; BC-ME-E-DEX (ME-loaded BC containing DEX).

#### 2.5.1. Transparency

Transparency of loaded BC-ME patches was assessed as previously described, with minor modifications [31,58]. The patches were transferred to a 24-well plate (Greiner, Nuertingen, Germany), and mean transmission at 650 nm was measured at five points located on the surface of the patches (Tecan Infinite^®^ M Nano, Tecan, Männedorf, Switzerland). Aliquots of deionized water of the same layer thickness as the patches were used as positive control. Experiments were carried out in triplicates, and results are expressed as mean ± SD.

#### 2.5.2. FF-TEM

For the TEM imaging and evaluation of the ME distribution within the BC network via freeze–fracture transmission electron microscopy (FF-TEM), sample pieces of ~1.5 mm × 1.0 mm × 0.5 mm were cut out of larger pieces of BC-ME with a scalpel. The samples were freeze–fractured and replicated as described earlier (Section 2.3.6). The freeze–fracture replicas were cleaned for 30 min at 70 °C in a solution of 4% (*w*/*v*) potassium hypochlorite and 30 min in sulfuric acid (50 vol%). The replicas were finally picked onto copper EM grids (400 mesh). Digital images were taken using a Philips CM120 electron microscope (Phillips, Eindhoven, Netherlands), operated at 120 kV. Images were taken with a 2K CMOS camera (F216, TVIPS GmbH, München, Germany).

### 2.6. In Vitro Strat-M^®^ Permeation and Anti-Inflammatory Activity

#### 2.6.1. Strat-M^®^ Membrane Permeation Testing

In vitro permeation testing of HC and DEX from BC-ME-HC and BC-ME-DEX was performed using vertical diffusion cells with a diffusion surface area of 1.77 cm^2^ and a receptor volume of 10.0 mL (Copley Scientific Limited, Nottingham, UK). To simulate permeation across human skin, Strat-M^®^ membrane (Merck KGaA, Darmstadt, Germany) was used. To ensure sink conditions, PBS pH 7.4 with addition of 20 vol% ethanol (Carl Roth) was used as the receptor medium [59,60]. The receiver compartment was tempered to 32 °C and magnetically stirred at 600 rpm (HDT 1000 Vertical Diffusion Tester, Copley Scientific Limited, Nottingham, UK). For permeation testing, BC-ME-HC and BC-ME-DEX patches were loaded as described earlier (Section 2.5). The loaded patches with a weight of 1.0 ± 0.1 g were placed in the donor compartment, and samples of 0.5 mL were collected from the receptor compartment at predetermined time intervals (t = 0, 0.5, 1, 2, 4, 8, 24, 32, 48, 72 h) and replaced with fresh and preheated receptor medium. In order to compare the API permeation with commercially available HC and DEX cream formulations, EBENOL^®^ 0.5% Creme (“EBENOL^®^”, Strathmann GmbH & Co. KG, Hamburg, Germany) and DEXAMETHASON Creme LAW; 0.05% (“DEXA-LAW”, Abanta Pharma GmbH, Plettenberg, Germany) were also evaluated. For the testing of these semisolid formulations, 1.00 g of cream, which was equivalent to the weight of loaded BC-ME patches, was transferred directly to the donor chamber. Finally, the release samples were lyophilized at −40 °C and 0.12 mbar (ALPHA 1-2 LD PLUS freeze-dryer, Martin Christ Gefriertrocknungsanlagen GmbH, Osterode am Harz, Germany), reconstituted with methanol (Carl Roth), and API content of the samples was determined by UHPLC. All experiments were performed in triplicate.

#### 2.6.2. API Release and Permeation from BC-ME

The anti-inflammatory activity of HC- and DEX-loaded BC-ME after release and permeation through an artificial skin barrier (Strat-M^®^ membrane) was investigated in a two-step test protocol. In the first step, a vertical diffusion test setup with human skin predictive Strat-M^®^ membranes as described earlier (Section 2.6.1) was used with the following modifications: RPMI 1640 medium (Sigma-Aldrich) was used as the receptor medium to ensure optimal conditions for subsequent cell culture experiments. The permeation test was performed for 72 h at 32 °C and 600 rpm stirring. The receptor medium after 72 h permeation that contained released HC and DEX from BC-ME patches was subsequently used in the second step to perform glucocorticoid activity testing (Section 2.6.3).

#### 2.6.3. Monocyte Incubations and Determination of TNFα Release

Human monocytes were isolated and incubated as described by Czapka et al. [61]. Briefly, leukocyte concentrates were prepared from freshly withdrawn peripheral blood of healthy human volunteers (age 18–65 years) provided by the Institute of Transfusion Medicine at the University Hospital Jena (Jena, Germany). Protocols for experiments with human blood cells were approved by the ethical commission of the Friedrich Schiller University Jena (Jena, Germany), and all methods were performed in accordance with the relevant guidelines and regulations. Peripheral blood mononuclear cells (PBMCs) were isolated using dextran sedimentation, followed by centrifugation on lymphocyte separation medium (Histopaque-1077, Sigma-Aldrich) and incubation in RPMI 1640 medium (Sigma-Aldrich) supplemented with 5 vol% heat-inactivated fetal calf serum (Sigma-Aldrich), 2 mM L-glutamine (Biochrom/Merck, Berlin, Germany), 100 U/mL penicillin and 100 µg/mL streptomycin (Sigma-Aldrich). The incubations were performed at 37 °C and under 5% CO_2_. After 1 h, non-adherent cells were removed from the culture flasks (Greiner, Nuertingen, Germany), and the monocyte-enriched PBMC fraction was transferred to 12-well plates (Greiner, Frickenhausen, Germany). Cells (1 × 10^6^ in 1 mL) were allowed to attach for 1.5 h and subsequently treated for 30 min with 10 µL of vehicle (RPMI medium) or RMPI medium obtained from Strat-M^®^ permeation test containing either released DEX or HC as described above. To include the technical replicates, the RMPI medium was pooled from three independent permeation tests per BC-ME sample. After 21 h of stimulation with 100 ng/mL lipopolysaccharide (LPS, Sigma-Aldrich), the cell-free supernatant was stored at −20 °C until determination of extracellular cytokine levels was performed by enzyme-linked immunosorbent assay (ELISA) according to manufacturer’s instructions (R&D Systems, Minneapolis, MN, USA).

### 2.7. Quantification of Glucocorticoids DEX and HC

HC and DEX content was quantified by UHPLC on an Agilent 1290 Infinity UHPLC coupled to a 6130 Single Quadrupol mass spectrometer (Agilent Technologies, Santa Clara, CA, USA). Chromatographical analysis was carried out on a Nucleodur C18 Gravity EC 50 × 2.0 mm, 1.8 µm (Macherey&Nagel, Düren, Germany) using the following gradient of water with 0.1% formic acid (eluent A) and acetonitrile (eluent B): flow: 1.0 mL/min; temperature: 30 °C; 0–4 min: 5–72% B; 4–4.5 min: 72–95% B; 4.5–5 min: 95% B; 5–5.5 min: 95–5% B; 5.5–6 min: 5% B. Signals were detected by ESI-MS in positive ionization mode, and UV signals were recorded at 242 nm and 246 nm for DEX and HC, respectively.

Quantification was accomplished by integration of the area under the curve using UV signals at the maximum excitation wavelength of the analytes with calibration curves ranging from 12.5 to 800 µM. For calibration, micronized DEX and HC (Euro OTC) dissolved in methanol (Carl Roth) served as standard for quantification.

## 3. Results and Discussion

### 3.1. Microemulsion Characterization

#### 3.1.1. Pseudo-Ternary Phase Diagram

**Figure 1:**
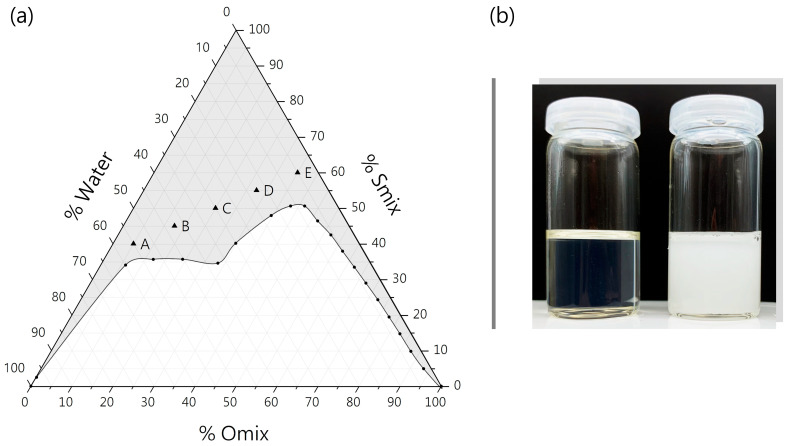
(**a**) Pseudo-ternary phase diagram of Smix, Omix and water phase, with gray area representing microemulsion region. (**b**) Transparent microemulsion (ME; left) turns turbid following addition of water due to formation of larger emulsion droplets.

In this work, MEs were chosen as a vehicle to solubilize the poorly water-soluble glucocorticoids HC and DEX in order to develop anti-inflammatory, dermal drug delivery systems based on BC. For the formulation of several ME, ingredients that in previous work showed sufficient solubilization capacity for hydrocortisone acetate, a structural analog of HC and DEX, were selected [48]. To map the ME region, a pseudo-ternary phase diagram was constructed by the water titration of surfactant and oil mixtures (Smix and Omix), whereas only clear, transparent and isotropic mixtures that formed fast and spontaneously were identified as MEs (Figure 1a). Further addition of water led to turbid emulsions, the coalescence of oil droplets and even phase separation (Figure 1b) [52].

The main region in which the ME formation could be observed was characterized by a surfactant content higher than ~40% that set interfacial tension low enough to allow relatively high amounts of oil (<~40%) and water (<~60%) to emulsify. High proportions of oil could only form isotropic mixtures with very low water content and vice versa, indicating the formation of micellar or inverse micellar solutions [42]. The ME region was found to be wide enough for investigating combinations of BC and MEs with several divergent compositions. Subsequently, five formulations (ME-A, ME-B, ME-C, ME-D, ME-E) that covered a wide range of Smix/Omix/Water ratios were selected.

#### 3.1.2. Electrical Conductivity and ME Microstructure

For the detailed characterization and identification of the ME microstructure, the determination of the electrical conductivity has proven to be a suitable tool [40,51,62]. The gradual dilution of surfactant-oil mixtures with the polar water phase leads to microstructural transitions which can be observed as characteristic changes in conductivity and allow the distinction of bicontinuous, oil-in-water (o/w) or water-in-oil (*w*/*o*) MEs [40,51].

As previous work suggests, conductivity values near zero indicate w/o microstructure, as the water droplets are in an isolated state and the conductivity of the continuous phase is fairly low due to the low polarity of the surfactant–oil mixture. With further dilution, the water droplets start to coalesce while forming interconnected polar channels, as indicated by a sudden increase in conductivity, thus signaling the transition point from w/o to bicontinuous ME microstructure. The conductivity increases linearly with further addition of water, as the number of polar channels increases. In the final phase, the increasing water content leads to the transition of a bicontinuous ME to an o/w ME, as indicated by the conductivity reaching a plateau, since a continuous water phase has formed surrounding isolated oil droplets [40,63,64].

**Figure 2:**
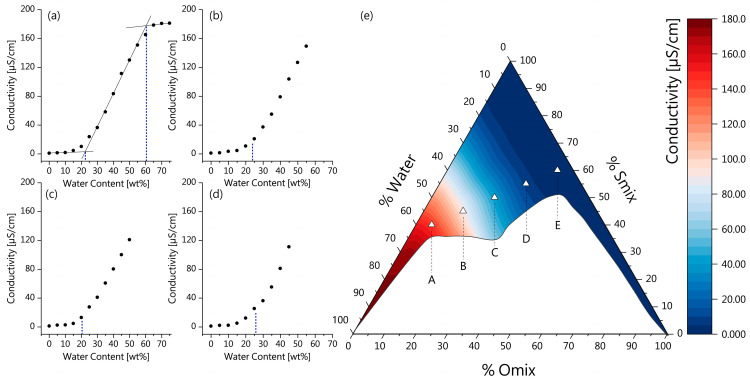
Characterization of ME formulations based on electrical conductivity; electrical conductivity of varying Smix/Omix mixtures as function of water content: Smix: (**a**) 95 wt%; (**b**) 85 wt%; (**c**) 75 wt%; (**d**) 65 wt%. (**e**) Conductivity of ME formulations based on their position in pseudo-ternary phase diagram. Error bars were omitted for clarity.

For this purpose, various mixtures of surfactant and oil were prepared, and the electrical conductivity was measured along the water dilution lines and plotted against the water content. Four dilution lines that cover the main ME area are shown in Figure 2a–d, as well as the conductivity transition related to the position of formulations in the pseudo-ternary phase diagram (Figure 2e). To determine the transition points from one ME type to another, the approximately linear parts of the conductivity plot were extrapolated, and their intersections were used to determine the water content at which the transition occurred, according to Zhang et al. [40], as shown in Figure 2a. The first transition point from w/o to bicontinuous MEs was observed for all dilution lines at a water content between 20 and 25 wt% (Figure 2a–d), which is in line with previous studies [40,64,65]. For the dilution shown in Figure 1a, a second transition point from bicontinuous to o/w MEs was observed at ~60 wt% water. Dilution lines of mixtures with higher oil content did not reach a conductivity plateau within the ME area, as the further addition of water led to the formation of conventional emulsions (see also Section 3.1.1).

For the five ME formulations, the conductivity values are shown in Table 2, together with their composition. Based on their conductivity values and the transition points at dilution lines crossing their positions in the pseudo-ternary phase diagram, the following ME types were identified: ME-E and ME-D were characterized as w/o ME, with ME-D approaching the transition point to a bicontinuous ME. For ME-C and ME-B, bicontinuous microstructure with different compositions is proposed. ME-A approaches the transition point from bicontinuous to o/w microstructure. The distinction of the five formulations allows further analysis of specific behavior in combination with BC, drug stability, permeation and activity.

#### 3.1.3. Physicochemical Characterization

The five selected ME formulations were further characterized with respect to the pH value, hydrodynamic diameter, refractive index, zeta potential and rheology (Table 3).

The pH value for all five MEs was found to be around pH 3.2. For the solubilization of HC and DEX, this pH is considered to be favorable since the pH stability optimum of HC was reported to be close to pH 3 [66], and the stability of DEX was observed to be higher under acidic conditions [67].

In general, the characterization of MEs via dynamic light scattering should only be applied to systems with discrete aggregates such as o/w or w/o MEs with a clear continuous phase [39]. For ME-A and ME-E that were identified as disperse ME in conductivity studies, Z-Ave values of 13.22 ± 1.54 nm and 11.24 ± 0.63 nm, respectively, were determined, which is in line with previous works that used similar surfactant–cosurfactant mixtures [68]. For the bicontinuous MEs (ME-B, ME-C, ME-D), Z-Ave values are also shown but should not be misinterpreted as droplet sizes since there are no droplet entities [64].

The zeta potential of all five ME formulations was observed to be close to zero mV, reflecting the neutral charge of the ME droplets, which can be attributed to the use of nonionic surfactants. These results tie well with previous studies using similar surfactant types [49,69].

The examination of ME rheology revealed viscosities ranging from 20.90 ± 0.17 to 78.08 ± 0.51 mPa·s and a linear correlation between shear stress and shear rate, intersecting with zero. This result indicates Newtonian flow behavior for all five ME formulations, which is characteristic of MEs and distinguishes the systems from liquid crystal phases with higher viscosity and pseudo-plastic flow behavior [70,71,72,73]. The viscosity of the investigated ME formulations increased from ME-A to ME-E which can be in part explained by the decreasing water content, since the viscosities of the selected Omix and Smix ingredients were significantly higher, such as 3000 mPa·s for Plurol Oleique^®^ CC 497 [74]. However, ME-D and ME-E showed very similar viscosities. A possible explanation might be that significant changes in viscosity are also indicative of microstructural changes at the transition points between o/w, bicontinuous and w/o ME microstructures [75,76]. Since ME-E and ME-D were both identified as w/o ME, the transition point from bicontinuous to w/o MEs has already been passed, the aqueous droplets are in an isolated state, and minor changes in continuous phase composition are unlikely to affect the ME viscosity significantly.

The thermodynamic stability of the ME formulations was evaluated via centrifugation and a freeze–thaw test. None of the samples showed signs of phase separation, aggregation or turbidity, indicating the thermodynamic stability of all samples.

In this work, the characterized MEs were chosen as vehicles to load BC with glucocorticoids HC and DEX. To simulate real-life conditions, the concentrations for the loading of HC and DEX into ME formulations were chosen based on commercial semisolid dermal formulations, with 0.5 wt% for HC and 0.05 wt% for DEX, whereas the lower DEX concentration is attributed to the higher potency of the glucocorticoid.

All five ME formulations were able to dissolve HC and DEX in less than 30 min at room temperature under moderate stirring, which makes the chosen ME formulations well suited for further investigation.

#### 3.1.4. In Vitro Cytotoxicity

**Figure 3:**
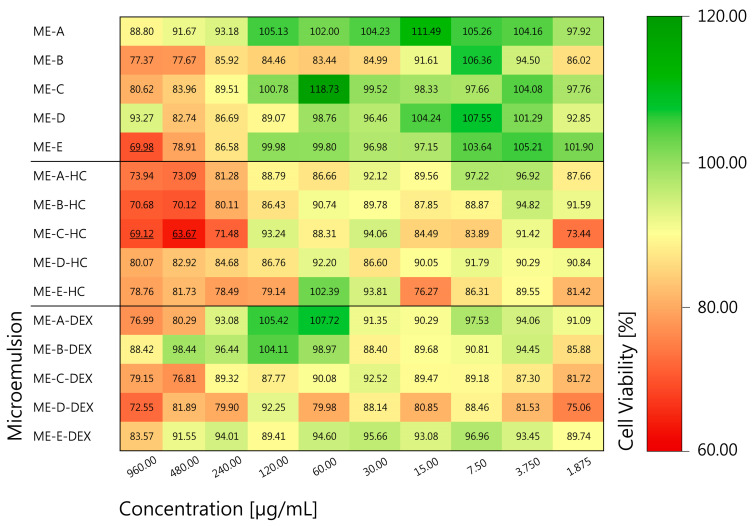
Mean cell viability of ME samples in MTT assay as percentage of untreated control cells. HaCaT cells were treated for 24 h with ME with concentrations ranging from 1.875 µg/mL to 960 µg/mL. Samples with viability < 70% are underlined. Samples were prepared in quadruplicates, and test was performed thrice. Standard deviations are omitted for clarity and can be found in Supplementary Materials Figures S1–S3.

The excellent biocompatibility of unmodified BC from *K. xylinus* strain DSM 14666 has already been demonstrated previously, e.g., in a standard MTT assay for macrophages [34,57] as well as for HaCaT keratinocytes in a luminometric ATP assay [77], and in a wound-healing scratch assay [34,57]. Therefore, the biocompatibility tests in this work focused on testing liquid ME samples ME-A to ME-E, as well as HC- and DEX-loaded MEs. HaCaT keratinocytes were chosen, as a dermal application on inflamed wounds was targeted for the BC-ME glucocorticoid combination. The mean cell viability at multiple concentrations as a percentage of untreated control cells is shown in Figure 3, while detailed results can be found in SI Figures S1–S3. According to DIN ISO 10993-5, a cell viability of ≥70% was regarded as non-toxic [55].

When considering the mean cell viability, the majority of the investigated samples and concentrations can be classified as non-toxic, with the exception of ME-E at 960 µg/mL as well as ME-C-HC at 960 µg/mL and 480 µg/mL. Apart from a few exceptions, the lowest cell viabilities were observed for the highest concentrations of 960 µg/mL and 480 µg/mL, respectively. Interestingly, ME formulations that contained HC led to slightly lower cell viabilities. We hypothesize that the higher initial concentration of 0.5 wt% HC in comparison to 0.05 wt% DEX may be responsible for these results. Overall, a note of caution is due here since all results showed fairly high standard deviations, which may be attributed to interactions between the excipients and the cell membrane because of structural similarities. Additionally, a high extracellular calcium level, present in this study due to the medium used, leads to reduced cell density and metabolic activity [78], which can result in a slower reduction in the MTT dye.

In general, most of the samples investigated can be classified as non-toxic even in high concentrations, which is an important prerequisite for the development and application of active wound dressings. To supplement these data, all of the excipients used in the formulation of the MEs in this work are currently listed in the quarterly updated FDA database “Inactive Ingredient Guide” (IIG [79]) as approved and incorporated in marketed products for topical use. In this context, extensive safety data were collected and evaluated by regulatory authorities.

#### 3.1.5. FF-TEM of ME Formulations

**Figure 4:**
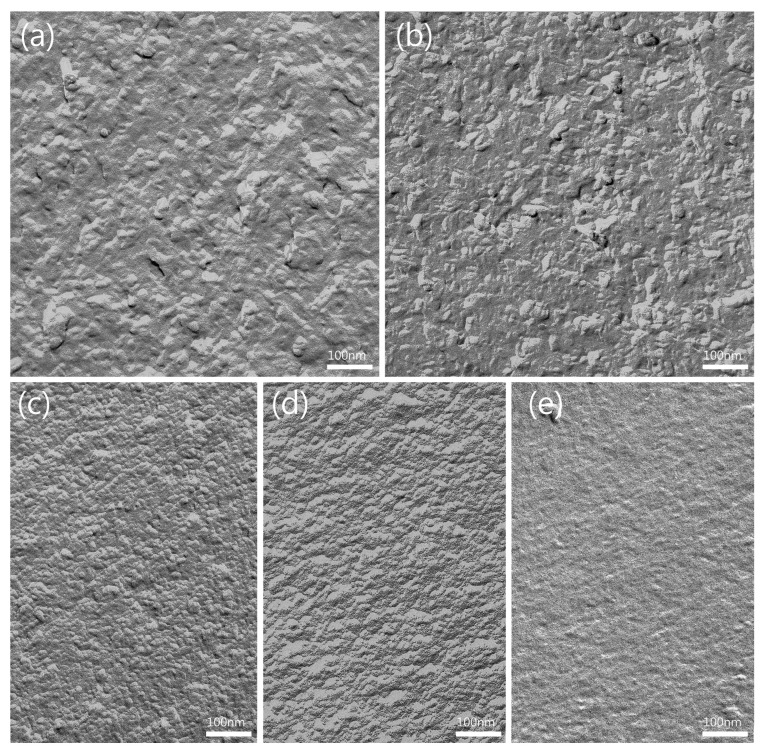
FF-TEM micrographs of (**a**) ME-A, (**b**) ME-B, (**c**) ME-C, (**d**), ME-D and (**e**) ME-E at 140,000× magnification. Images were rotated with 35° shadowing orientated from bottom to top.

For further understanding and detailed analysis of the ME microstructure, all five ME formulations were imaged by FF-TEM (Figure 4). The use of this method for imaging MEs has been widely reported and minimizes the risk of artifact formation during sample treatment, especially for bicontinuous and w/o MEs [38,39]. All images show a granular structure and thus evidence of the colloidal structure of the samples. ME-A, which has been identified as an o/w ME based on conductivity, shows spherical and non-spherical structures that appear to be smaller than 50 nm and are likely an imaging of the dispersed oil phase (Figure 4a). The micrograph of bicontinuous ME-B and ME-C displays mainly non-spherical structures and a homogenous granular structure, which probably indicates the bicontinuous coexistence of both phases in interconnected channels, with both MEs presenting signs of a presumably weak, directional arrangement (Figure 4b,c). ME-D and ME-E, both identified as w/o type, display a homogenous granularity with occasional smooth areas, indicating dispersed water droplets (Figure 4d,e). A possibly directional arrangement as seen in the bicontinuous ME samples is no longer visible. The overall appearance of the micrographs is in line with former works and confirms the existence of ME structure and the microstructure identification based on conductivity [80].

### 3.2. Stability Testing

#### 3.2.1. Storage Stability

To detect and quantify the potential degradation of HC and DEX in all five ME formulations over time, the storage stability was investigated at three different storage conditions: 4 °C (refrigerator), 21 °C (room temperature) and 40 °C (accelerated conditions), with sampling points at 0, 30 and 90 days and with the addition of EDTA as a metal complexing agent to investigate degradation due to trace metal ions.

**Figure 5:**
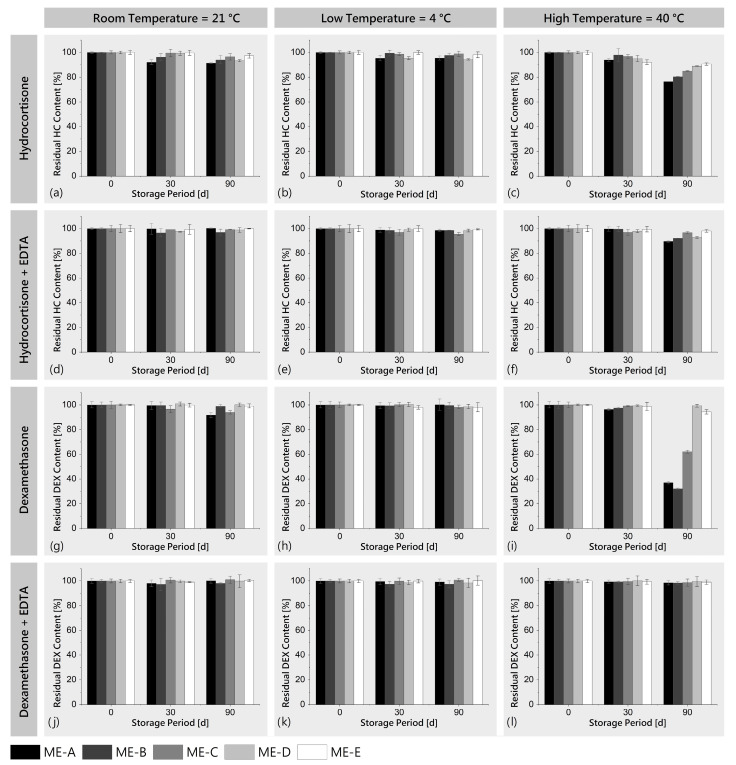
Residual drug content of ME-HC (**a**–**f**) and ME-DEX (**g**–**l**) after storage at 4 °C, 21 °C and 40 °C (mean ± SD, *n* = 3) for 30 and 90 days as percentage of initial content and with addition of metal complexing agent EDTA to investigate its effect on degradation caused by trace metal ions.

The tests showed only slight degradation of HC and DEX at 21 °C and 4 °C, even over the longest testing period of 90 days, without any sample showing a residual content of less than 90% of control. In contrast to this, accelerated storage at 40 °C led to stronger degradation effects for specific samples, especially after 90 days of storage. For HC, the degradation after 90 days showed a correlation with the water content of the ME and was strongest for ME-A with 76.46 ± 0.25% of control (Figure 5c). Interestingly, the degradation of DEX was found to be even stronger (Figure 5i), in particular for ME-A (37.10 ± 1.09%), ME-B (32.19 ± 0.56%) and ME-C (62.08 ± 1.24%) which contained higher ratios of water. Remarkably, the addition of the metal complexing agent EDTA exhibited a strong protective effect, with none of the samples showing a residual content of less than 90% for HC (Figure 5d–f) and less than 98% for DEX (Figure 5j–l). For most pharmaceuticals, >90% potency at the end of the storage period is a standard shelf-life specification based on USP monographs [81]. For the formulations tested in this study, the addition of EDTA can be used here as a measure to ensure that the API potency does not fall below this limit.

With regard to the literature, the main degradation pathways of glucocorticoids in the presence of water and oxygen containing a C-17 dihydroxacetone side chain such as HC and DEX are proposed to be via the oxidative and hydrolytic reactions of the C-17 side chain, with oxidative degradation being strongly catalyzed by trace metal ions such as copper or iron [82,83,84]. The complexation of these trace metal ions by chelating agents such as EDTA is a well-described means to inhibit the degradation of these glucocorticoids, which ties well with the observed results [82,85,86]. In conclusion, the generally good stability of HC and DEX can be increased by controlled storage at lower temperatures or the addition of metal complexing agents such as EDTA. Although we hypothesize that a combination of controlled, low-temperature storage with EDTA leads to long-term drug stability which is beneficial for a stable and effective product, future studies should evaluate the chemical stability for periods longer than 90 days, the influence of possible degradation products on the biocompatibility and toxicity [82,83,84] of the formulations and the effects of excipients on drug permeation and wound healing.

#### 3.2.2. Sterilization Stability

**Figure 6:**
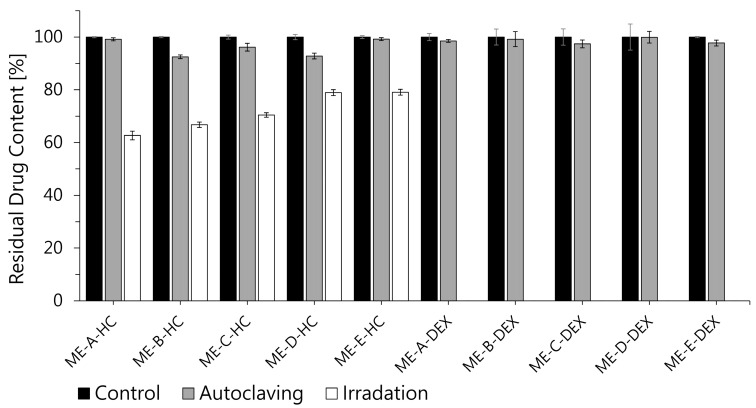
Residual drug content of ME-HC and ME-DEX samples after sterilization via autoclaving and irradiation (mean ± SD, *n* = 3). While autoclaving generally resulted in only minor degradation, irradiation of ME-HC samples led to reduction in drug content to range of 60% to 80% of the control. DEX was less resistant to irradiation and no longer detectable in irradiated samples.

To inactivate microbial contaminants and prevent secondary infection in chronic wounds, applied dressings are usually processed by thermal, chemical or irradiation sterilization procedures [87]. The influence of this processing on the dressing material, as well as on incorporated APIs, must be carefully analyzed to ensure that physical and chemical degradation remains within tolerable limits. For this purpose, e-beam irradiation and autoclaving at 121 °C and 2 bar were selected to investigate their influence on the chemical stability of HC and DEX in ME samples, since the stability of BC as the dressing and carrier material toward these procedures has been shown in previous studies [31,56]. Figure 6 shows the drug content of HC- and DEX-loaded MEs after autoclaving and e-beam sterilization as a percent of unsterilized control. Both glucocorticoids showed only slight degradation after autoclaving, with none of the samples exhibiting a drug content of less than 90% of the control.

Interestingly, e-beam irradiation had a higher and more variable impact on the measured drug content. The HC content of sterilized samples was found to be within 62.67 ± 1.66% (ME-A) and 79.06 ± 1.09% (ME-E), with a trend toward higher degradation of water-rich, bicontinuous systems (ME-A, ME-B, ME-C) and higher stability of w/o systems (ME-D, ME-E), similar to the results obtained from storage stability testing. This fits well with the results of previous studies, which reported higher radiostability of the glucocorticoid prednisolone when formulated in o/w MEs, on the one hand, and protective effects of non-ionic surfactants on prednisolone, on the other hand [88,89].

Surprisingly, DEX was no longer detectable in irradiated ME samples. Although these findings seem to differ from the results of Marciniec et al., who reported only a slight and comparable degradation of solid-state HC and DEX after e-beam radiation (none less than 97% of control) [90], one has to keep in mind that the presence of water in liquid formulations can have a strong impact on the API decomposition, mainly due to the radiolytic formation of reactive species (•OH, H•, e_aq_^−^, H_2_O_2_) [91,92]. Although the degradation of glucocorticoids in the presence of water has so far only been investigated for gamma-irradiation, it seems likely that the decomposition mechanisms are to some extent comparable to e-beam irradiation, since gamma-radiation has been shown to form similar reactive species in the presence of water [93]. In this regard, previous work has proposed the oxidative cleavage of the C-17 side chain to C-17 ketone and the conversion of C-11 alcohol to ketone as major degradation paths for glucocorticoids [94] and more recently •OH radical oxidation, •OH radical substitution and the direct decomposition of the molecule specifically for DEX [93].

It can be concluded that autoclaving could be a suitable sterilization method for ME-HC- and ME-DEX-loaded wound dressings based on BC regarding chemical stability, although whether the method leads to the formation of toxic degradation products needs to be tested. E-beam sterilization cannot be recommended due to the partial (HC) or complete (DEX) decomposition of incorporated API. Nevertheless, further work should be carried out to confirm these results for the combination of ME-HC and ME-DEX with BC.

### 3.3. Loading, Permeation and Anti-Inflammatory Activity

#### 3.3.1. Loading Behavior

The absorption loading of native BC patches with all five MEs revealed that all samples regained over 90% of their initial weight after 72 h of loading. In comparison to native BC patches, the ME-loaded patches appeared transparent in varying intensities (see also Section 3.3.2), which allowed us to assess and confirm the homogenous distribution of the MEs in the BC patches. This could be expected for o/w MEs based on the similarity of the continuous phase and the hydrophilic cellulose network, especially with regard to previous works investigating nanoemulsions [29,31]. It is remarkable that the loading with bicontinuous and particularly w/o-type MEs seems to work comparably well, with regard to the lipophilic continuous phase. It may be assumed that the polar head groups of the amphiphilic surfactant molecules surround the hydrophilic cellulose fibers, mainly characterized by OH groups in a comparable manner to water droplets. This could be a consequence of the extremely low interfacial tension and the high flexibility of the surfactant layer and correlates with the characteristic ability of MEs to solubilize high amounts of both hydrophilic and lipophilic constituents [39,44]. It is strongly recommended that further research gathers evidence to confirm or falsify this hypothesis by focusing on the molecular and physicochemical interaction between ME systems and BC.

#### 3.3.2. Transparency

**Figure 7:**
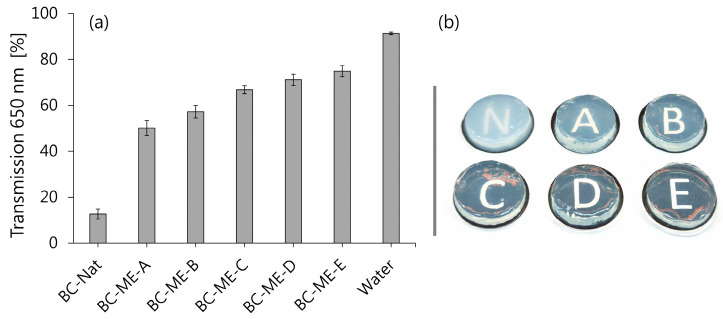
(**a**) Transparency of native BC and BC-ME: transmission at 650 nm (mean ± SD; *n* = 3). (**b**) Image of native BC and BC-ME patches with visible differences in transparency.

While BC normally appears white to translucent, it could be observed that the ME loading of BC led to an increase in optical transparency, which was subsequently quantified spectrophotometrically (Figure 7). While for native BC a relatively low transparency of 12.70 ± 2.17% was found, there was a remarkable increase in transparency for BC-ME, ranging from 50.12 ± 3.27% for BC-ME-A, up to 74.94 ± 2.43% for BC-ME-E. Interestingly, this increase in transparency correlated with decreasing water content and, more importantly, with the rising refractive index of the ME formulations (Section 3.1.3). As suggested previously by Bellmann et al. [58], the difference in transparency could be explained by the convergence of refractive indices of incorporated fluid and matrix material. The authors observed similar effects for loaded BC with glycerol/urea water mixtures of different compositions and hypothesized, with reference to Wildner et al. [95], that the matching refractive index led to minimized light scattering and thus increased transparency. Our study provides further evidence for this hypothesis. Furthermore, for the dermal application, the increased transparency is of considerable benefit, as it allows inspection of wounds and treated skin areas during the application, without the removal of the wound dressing.

#### 3.3.3. FF-TEM of BC-ME

**Figure 8:**
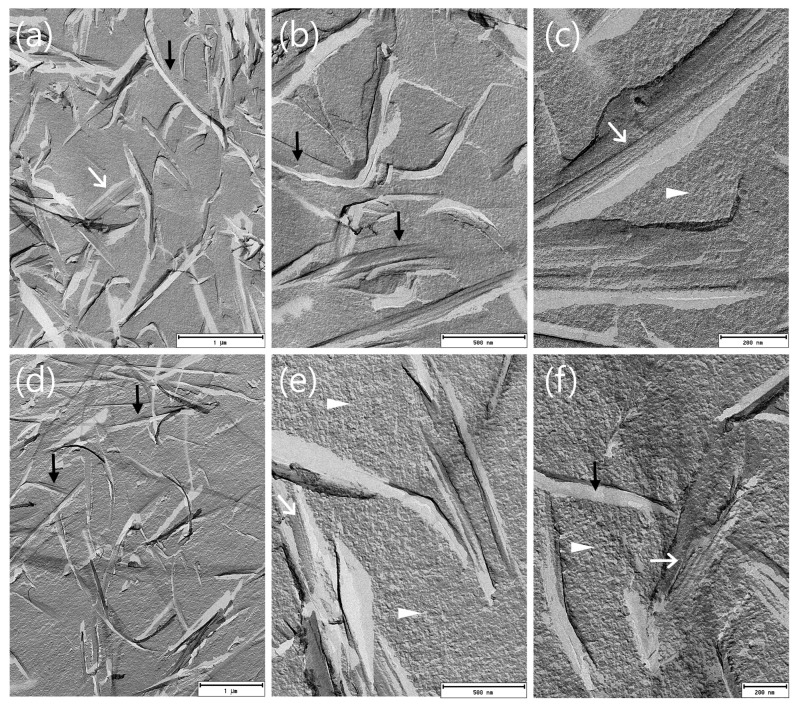
FF-TEM micrographs of BC-ME-A (**a**–**c**) and BC-ME-B (**d**–**f**) at different magnifications. The BC fibers lead to ribbon-like structures in the replica (black arrows), which merge directly into the homogenous, granular ME structure with convex and concave fracture surfaces (white triangles), indicating a homogenous distribution of the MEs between the cellulose fiber network. Regular structures (white arrows), due to the layered assembly of smaller cellulose protofibrils, are partly visible in the cellulose ribbons. Images were rotated with 35° shadowing orientated from bottom to top.

To evaluate the distribution of incorporated MEs in the BC network, BC-ME-A and BC-ME-B were imaged by freeze–fracture-replication transmission electron microscopy (FF-TEM) (Figure 8), analog to the ME samples (see also Section 3.1.5). The images show prominent, ribbon-like structures in the range of 100 nm thickness, depicting the cellulose fibers (Figure 8a,d). The granular ME structures seem to be homogenously distributed between the fibers, without visible aggregation at the fibers or cavities, which matches well with the neutral zeta potential of the ME systems. Furthermore, it seems likely that the thermodynamic stability as a consequence of the extremely low interfacial tension of MEs in general is beneficial for the homogenous distribution inside the cellulose network.

For the incorporation of APIs in BC using ME, the homogenous distribution as shown is an important prerequisite for a predictable and uniform loading process. These findings furthermore support the suitability of MEs for the incorporation of lipophilic APIs into BC wound dressings.

**Figure 9:**
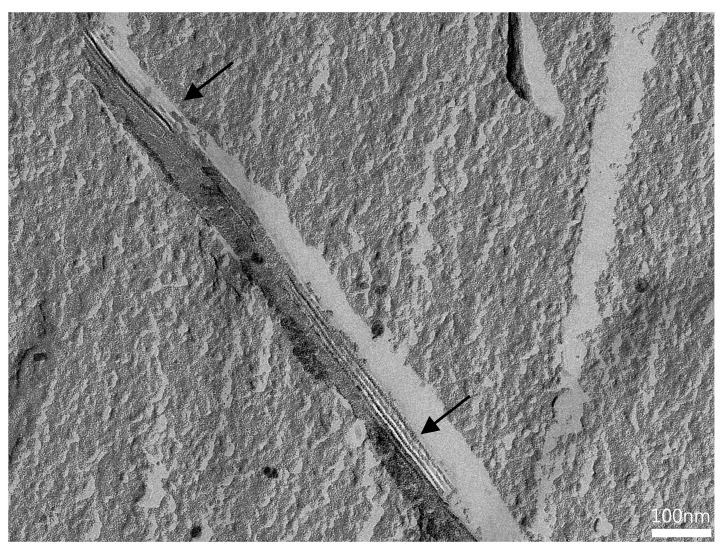
FFT-filtered FF-TEM image of regular structures at fracture surface due to layered assembly of cellulose protofibrils (black arrows). Distance between protofibrils was around 9.5 nm (scalebar 100 nm; see Supplementary Materials).

To the best of our knowledge, Figure 9 shows the first FF-TEM image of the hierarchical structure of ~ 80 × 4 nm BC ribbon-like microfibrils (*K. xylinus*) and their assembly by smaller protofibrils, allowing a very interesting insight into the BC ribbon nanostructure. Important to mention is the recent work of Babi et al. [96], who published TEM images of the BC supramolecular structure as well. Nevertheless, the FF-TEM technique in combination with plunge freezing is likely to better preserve the native microstructure, as well as show the structure of never-dried BC, in contrast to the drying involved in other imaging techniques, such as atomic force microscopy (AFM) and TEM.

#### 3.3.4. In Vitro Permeation

**Figure 10:**
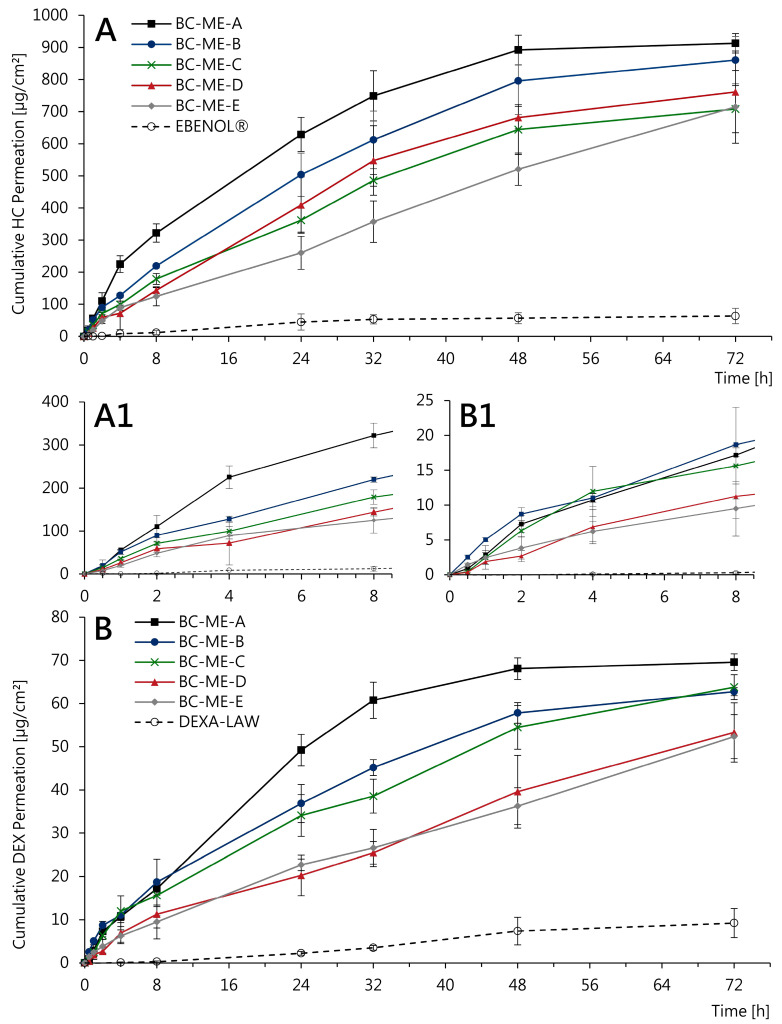
Cumulative permeation profiles of HC (**A**,**A1**) and DEX (**B**,**B1**) from BC-ME and conventional semisolid formulations determined in a vertical diffusion cell setup using synthetic, skin-mimicking Strat-M^®^ membranes at 32 °C (mean ± SD, *n* = 3).

With regard to the dermal application, the API permeation of incorporated glucocorticoids from BC-ME-DEX and BC-ME-HC was studied in a vertical diffusion cell setup using Strat-M^®^ membranes as synthetic skin models. These multilayered membranes have been shown to reliably predict the permeation of lipophilic and hydrophilic APIs with low variability and high correlation to human skin [97,98,99,100]. Besides the novel BC-based systems, marketed semisolid formulations HC-0.5 and DEX-0.05 with comparable dosage strength were tested in the same setup for comparison. The dependence of the cumulative amount of the permeated API on time for up to 72 h is shown in Figure 10. The drug permeation of HC, which is about a factor of 10 higher, correlates well with the drug concentrations used, whereby DEX is concentrated 10 times lower (0.05%) than HC (0.5%) due to the higher potency of the glucocorticoid.

For both APIs, the permeation profile was highly affected by the selected ME formulation. For HC, BC-ME-A showed a higher permeation than all other formulations, slowing down after 8 h and reaching a plateau after 48 h (Figure 10A). In contrast, the permeation profile of BC-ME-E exhibits linear characteristics with constant API permeation and does not reach a plateau during the 72 h testing period. Although less API is permeated in total, it is likely that permeation would have been going on after 72 h, stagnating at a later time point. The permeation profiles of the three remaining ME formulations appear to fall between those mentioned before. Although a successive transition in relation to the ME microstructure can be suspected, this cannot be clearly confirmed based on the standard deviation of the available results. Interestingly, despite the comparable drug concentration and dosage tested, the commercial semisolid formulation EBENOL^®^ resulted in lower API permeation, with less than one-tenth of the amount of drug permeated from the BC-ME samples after 72 h. Similar results are observed in the permeation profiles of the DEX samples (Figure 10B), with BC-ME-A standing out clearly from the remaining formulations. Furthermore, API permeation from the semisolid formulation DEXA-LAW is again low in comparison.

A possible explanation for the different permeation characteristics can be found in the rheological properties of the incorporated ME formulations. ME-A, which features low viscosity and a high water content, exhibits fast and strong drug permeation likely to be the consequence of a burst release which is characteristic of BC-based delivery systems using low viscous formulations [31,34,57]. However, it seems likely that with the increased viscosity of ME-D and ME-E, the drug diffusion inside the cellulose network is slowed down, resulting in sustained release and thus continuous and more linear permeation. This correlates fairly well with the results of Alkhatib et al., who described the long-term release of octenidine from BC by the incorporation of poloxamer and consequent gelation inside the cellulose matrix [28]. The selection of a specific ME formulation can therefore be used as a tool to control drug permeation from BC-based wound dressings and delivery systems and adapt it, e.g., according to standard wear times.

The most striking result to emerge from the data is the remarkably higher glucocorticoid permeation compared to conventional semisolid formulations of the same dosage over 48 h to at least 72 h. The strong API permeation is not surprising, regarding the commonly known facilitating effects of MEs on drug penetration and permeation [41,42,44]. Additionally, the excipient diethylene glycol monoethyl ether (Transcutol^®^ P), which was included in all five ME formulations and is a commonly used permeation and penetration enhancer, can be held responsible for this positive effect to a certain degree [101,102]. However, it is fundamental to note that the continuous API permeation over 72 h is made possible by the BC network acting as a matrix material and thus allowing a clinically highly relevant long-term application over up to 3 days. In particular, inflamed wounds and dermatoses such as pyoderma gangraenosum could benefit significantly from a combination of the wound-healing-promoting properties of BC dressings and the high and controllable permeation of APIs such as the glucocorticoids HC and DEX.

#### 3.3.5. Anti-Inflammatory Activity

**Figure 11:**
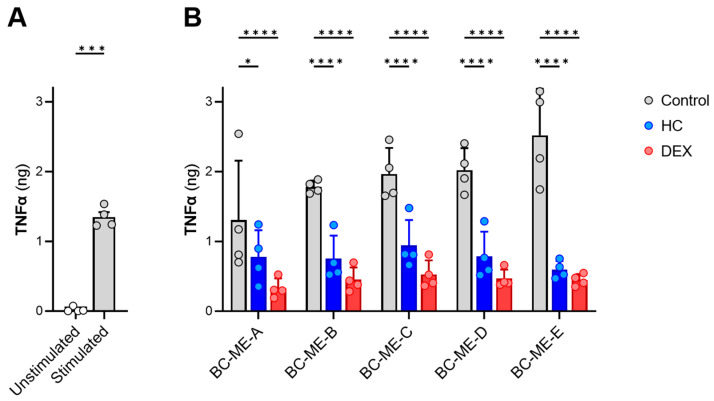
Effects on TNFα release from human monocytes after 21 h as determined by ELISA. (**A**) Stimulation of TNFα release from human monocytes by LPS versus vehicle-treated cells (unstimulated). (**B**) Downregulation of TNFα in LPS-stimulated human monocytes by HC and DEX after release from BC-ME and permeation through Strat-M^®^ membranes compared to BC-ME without API (control). Data are shown as mean ± SD from four different donors. Statistical analysis was performed by two-tailed paired *t* test (**A**) or RM two-way ANOVA (**B**) with Dunnett’s multiple comparisons test (single pooled variance). * *p* < 0.05; *** 0.0005; **** 0.0001.

To confirm the drug permeation and anti-inflammatory activity of HC and DEX BC-ME-HC and BC-ME-DEX, a two-part test design was used. The release and drug permeation of HC and DEX were conducted in vitro in a vertical diffusion cell setup, using Strat-M^®^ membranes as synthetic skin models, to obtain vertical diffusion cell receptor media samples potentially containing the APIs. Next, the obtained receptor media samples were investigated regarding their effect on suppressing the release of the pro-inflammatory cytokine TNFα in LPS-stimulated monocytes, as described by Czapka et al. [61]. As shown in Figure 11, all five formulations led to the significant downregulation of TNFα in comparison to the control without APIs, thus demonstrating their anti-inflammatory activity in vitro and indirectly confirming API permeation from BC-ME-DEX and BC-ME-HC through the synthetic skin model. This anti-inflammatory activity is particularly significant for the application as wound dressings for inflamed wounds and dermatoses, such as pyoderma gangraenosum, where managing inflammation is crucial to promote healing and prevent further tissue damage [15,16]. The ability of the BC-ME dressings to effectively deliver glucocorticoids suggests great potential for this combination to modulate the inflammatory response in wound environments. The downregulation of TNFα, which is strongly associated with impaired wound healing [103,104], demonstrates the anti-inflammatory effect but furthermore indicates that the formulation of the APIs in BC-ME wound dressings does not negatively impact glucocorticoid efficacy. Along with the sustained and high API permeation, the developed combination could lead to improved healing outcomes involving reduced pain and patient discomfort, highlighting the clinical relevance of anti-inflammatory drug delivery in the context of advanced wound management. The biological, anti-inflammatory activity of glucocorticoid-loaded BC-ME was thus successfully demonstrated in this proof of concept.

## 4. Conclusions

Five different MEs were developed as loading vehicles to incorporate the poorly water-soluble glucocorticoids HC and DEX into the hydrophilic network of BC for the topical application of the patches as anti-inflammatory wound dressings or drug delivery systems. After the characterization of the ME microstructure and FF-TEM imaging, all five ME formulations showed suitability to dissolve HC and DEX in therapeutically relevant concentrations, as well as biocompatibility for most concentrations in an MTT assay using HaCaT cells. Autoclaving was found to be a more appropriate sterilization method than e-beam irradiation, which led to API degradation to different extents. Still, further testing is required to ensure that no toxic degradation products are formed during autoclaving. The formulations exhibited good storage stability over 90 days, which could be further enhanced by the addition of EDTA as a metal complexing agent and is an important prerequisite for further product development and transfer.

Most importantly, BC patches could be successfully loaded with the ME formulations in a simple and fast procedure. Surprisingly, the loading behavior of the hydrophilic cellulose network was comparable for o/w, bicontinuous and even w/o MEs which requires further investigations to understand the interactions of MEs with the BC network. A homogenous distribution of o/w and bicontinuous MEs in BC could be demonstrated via FF-TEM imaging. The ME-loaded BC exhibited increased optical transparency, which correlated with the refractive index of the individual ME formulations and could be a beneficial characteristic for the optical assessment of a wound or skin area.

The results of in vitro permeation testing using Strat-M^®^ membranes as synthetic skin models revealed considerably higher drug permeation in comparison to commercial semisolid HC and DEX formulations. Depending on the selected ME formulation, the permeation profile could be controlled toward a continuous permeation for up to 72 h, providing the opportunity of long-term application with reduced dressing change frequency. The drug permeation and anti-inflammatory activity of released and permeated glucocorticoids were finally investigated in vitro by the determination of the effect on TNFα release in LPS-stimulated monocytes, successfully demonstrating the pharmacological activity and providing preliminary evidence for the effectivity of the technology regarding a dermal application.

The results obtained are very encouraging. For the first time, ready-formulated MEs could be incorporated into BC, a biomaterial well known to support wound healing, to develop stable and well-performing topical anti-inflammatory drug delivery systems for the treatment of inflamed dermatoses and wounds. The technology presented in this study has the potential to enable new treatment strategies, contribute to the effective management of skin conditions such as pyoderma gangraenosum and significantly improve patients’ quality of life. Further research should focus on making these promising results accessible for product development and translating them into clinical practice.

## Figures and Tables

**Table 1:** Composition of the prepared MEs.

Microemulsion	Smix ^1^ [wt%]	Water [wt%]	Omix ^2^ [wt%]
ME-A	40.0	55.0	5.0
ME-B	45.0	42.5	12.5
ME-C	50.0	30.0	20.0
ME-D	55.0	17.5	27.5
ME-E	60.0	5.0	35.0

^1^ Labrasol^®^ + Transcutol^®^ P, 5:1 ratio. ^2^ Plurol Oleique^®^ CC 497 + Labrafil^®^ M 1944 CS, 1.4:1 ratio.

**Table 2:** ME formulations with corresponding compositions and electrical conductivity (Mean ± SD).

Microemulsion	Smix [wt%]	Water [wt%]	Omix [wt%]	Conductivity [µS/cm]
ME-A	40.0	55.0	5.0	142.07 ± 0.98
ME-B	45.0	42.5	12.5	89.86 ± 0.90
ME-C	50.0	30.0	20.0	38.79 ± 0.76
ME-D	55.0	17.5	27.5	7.04 ± 1.92
ME-E	60.0	5.0	35.0	3.65 ± 2.75

**Table 3:** Characterization of unloaded ME formulations (Mean ± SD).

Microemulsion	pH	Refractive Index	Z-Ave [nm]	Zeta Potential [mV]	Dynamic Viscosity [mPa·s]
ME-A	3.16 ± 0.02	1.3938	13.22 ± 1.54	0.129 ± 0.470	20.90 ± 0.17
ME-B	3.18 ± 0.04	1.4115	6.98 ± 0.90	−0.081 ± 0.223	44.70 ± 0.20
ME-C	3.20 ± 0.06	1.4275	3.72 ± 0.09	0.169 ± 0.533	69.05 ± 0.66
ME-D	3.30 ± 0.02	1.4432	7.19 ± 0.18	0.044 ± 0.525	77.13 ± 0.86
ME-E	3.25 ± 0.06	1.4560	11.24 ± 0.63	0.010 ± 0.454	78.08 ± 0.51

## Data Availability

The data presented in this study are available in this article and Supplementary Materials.

## Supplementary Materials

The following supporting information can be downloaded at https://www.mdpi.com/article/10.3390/pharmaceutics16040504/s1, Figure S1: Mean cell viability of ME samples without glucocorticoids in MTT assay as percentage of untreated control cells (mean ± SD); Figure S2: Mean cell viability of HC-loaded ME samples in MTT assay as percentage of untreated control cells (mean ± SD); Figure S3: Mean cell viability of DEX-loaded ME samples in MTT assay as percentage of untreated control cells (mean ± SD); Figure S4 (a): FFT-filtered FF-TEM image of regular structures at fracture surface due to layered assembly of cellulose protofibrils. (b) Measurement of repetition distance in FFT image.

## Abbreviations and Terms

AFM	atomic force microscopy
API(s)	active pharmaceutical ingredient(s)
BC	bacterial cellulose
DEX	dexamethasone
DEXA-LAW	“DEXAMETHASON Creme LAW, 0.05%”, commercial dexamethasone cream
DMEM	Dulbecco’s Modified Eagle Medium
DSMZ	Deutsche Sammlung von Mikroorganismen und Zellkulturen (German Collection of Microorganisms and Cell Cultures)
EBENOL^®^	“EBENOL^®^ 0.5% Creme”, commercial hydrocortisone cream
EDTA	ethylene diamine tetraacetic acid (sodium salt dihydrate)
ELISA	enzyme-linked immunosorbent assay
FCS	fetal calf serum
FFT	fast Fourier transform
FF-TEM	freeze–fracture transmission electron microscopy
GR	glucocorticoid receptor
GRC	glucocorticoid receptor complex
GRE	glucocorticoid response element
HaCaT	human epidermal keratinocyte cell line
HC	hydrocortisone
HSM	Hestrin–Schramm culture medium
IL	interleukin
*K. xylinus*	*Komagataeibacter xylinus*
LPS	lipopolysaccharide
ME(s)	microemulsion(s)
MTT	3-(4,5-dimethylthiazol-2-yl)-2,5-diphenyltetrazolium bromide
NFκB	nuclear factor κB
Omix	oil phase mixture
PBMC	peripheral blood mononuclear cells
RH	relative humidity
rpm	revolutions per minute
RPMI	RPMI 1640 cell culture medium
Smix	surfactant/cosurfactant mixture
TNFα	tumor necrosis factor alpha
