# Supplementary material for: Exploring Microemulsion Systems for the Incorporation of Glucocorticoids into Bacterial Cellulose: A Novel Approach for Anti-Inflammatory Wound Dressings
Source: Pharmaceutics. 2024 Apr 7;16(4):504. doi: 10.3390/pharmaceutics16040504 (PMC11054342; doi:10.3390/pharmaceutics16040504)
Supplement: Supplementary file 1 [file pharmaceutics-16-00504-s001.zip › pharmaceutics-2890228-supplementary.pdf]

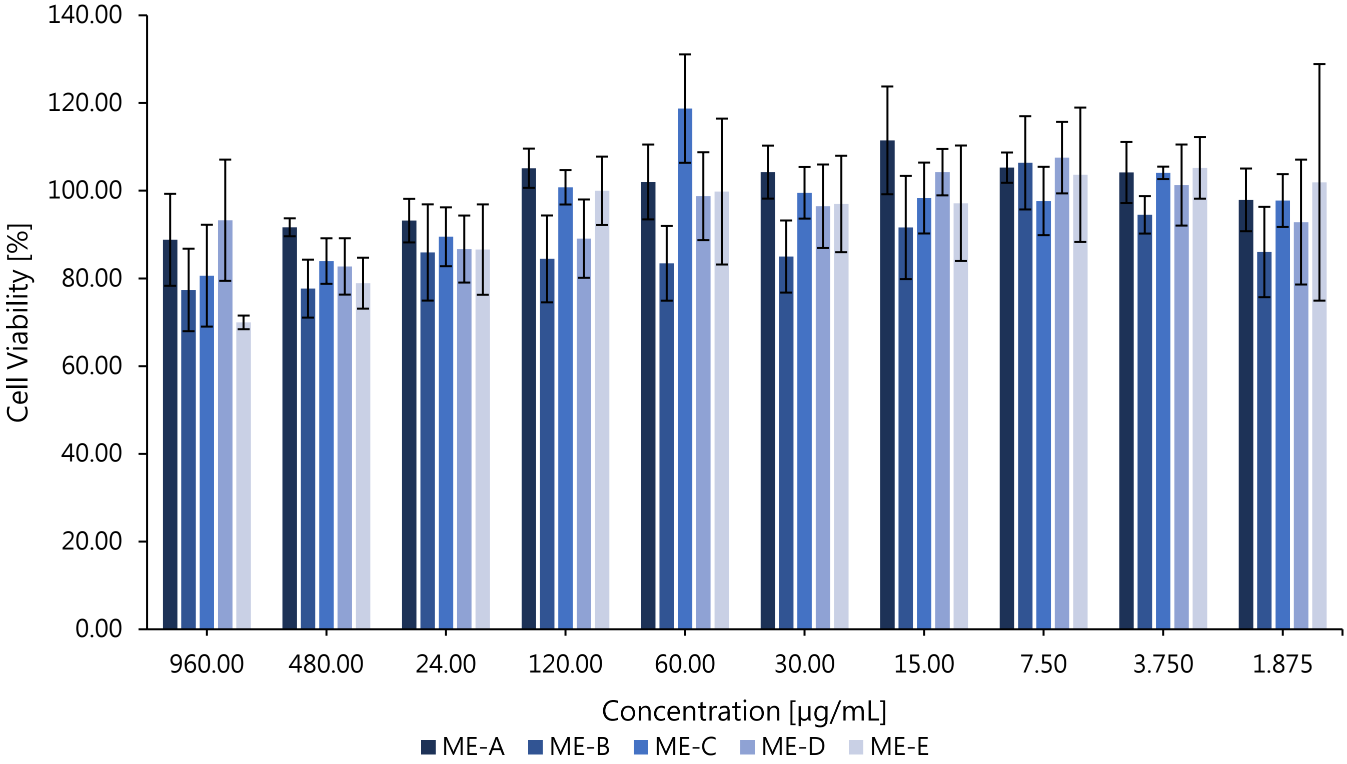

Figure S1. Mean cell viability of microemulsion (ME) samples without glucocorticoids in MTT (3-(4,5-dimethylthiazol-2-yl)-2,5-diphenyltetrazolium bromide) assay as percentage of untreated control cells. Human keratinocyte (HaCaT) cells were treated for 24 h with ME with concentrations ranging from 1.875 µg/mL to 960 µg/mL. Samples were prepared in quadruplicates and the test was accomplished thrice. Data are shown as mean ± SD.

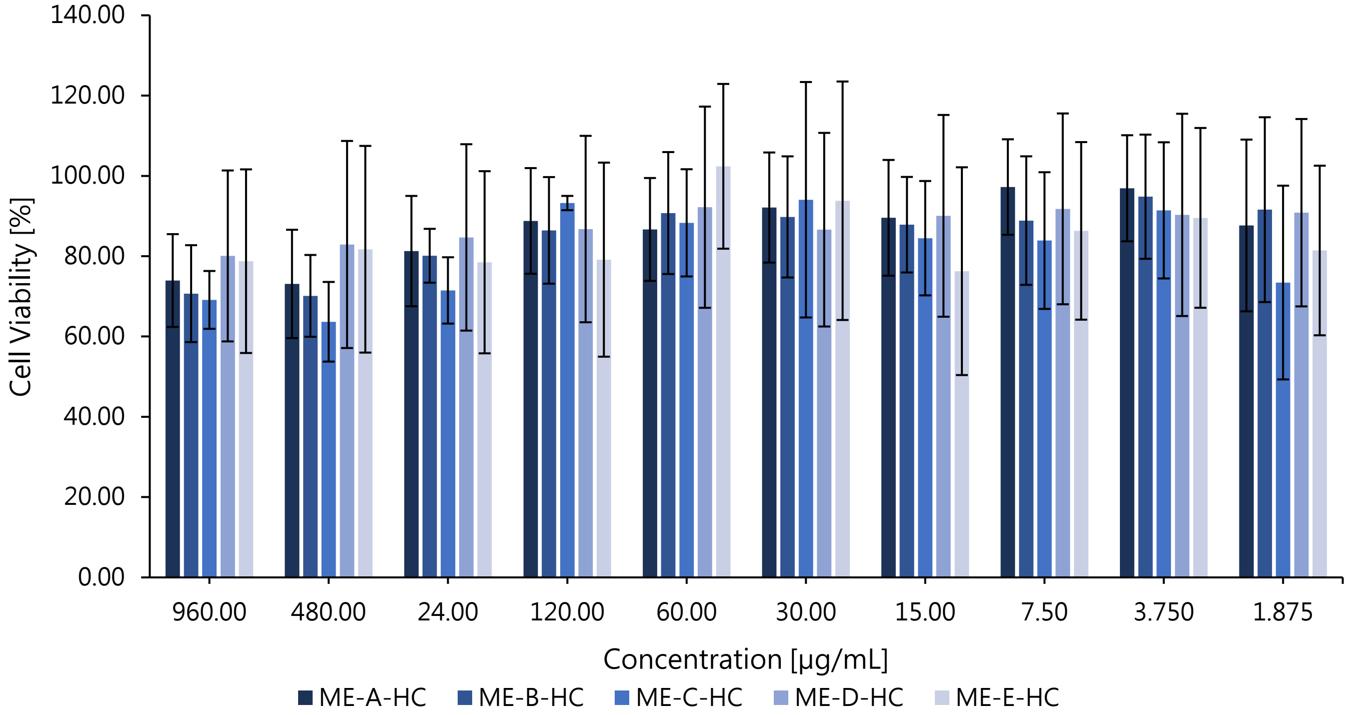

Figure S2. Mean cell viability of hydrocortisone loaded ME samples in MTT assay as percentage of untreated control cells. HaCaT cells were treated for 24 h with ME with concentrations ranging from 1.875 µg/mL to 960 µg/mL. Samples were prepared in quadruplicates and the test was accomplished thrice. Data are shown as mean ± SD.

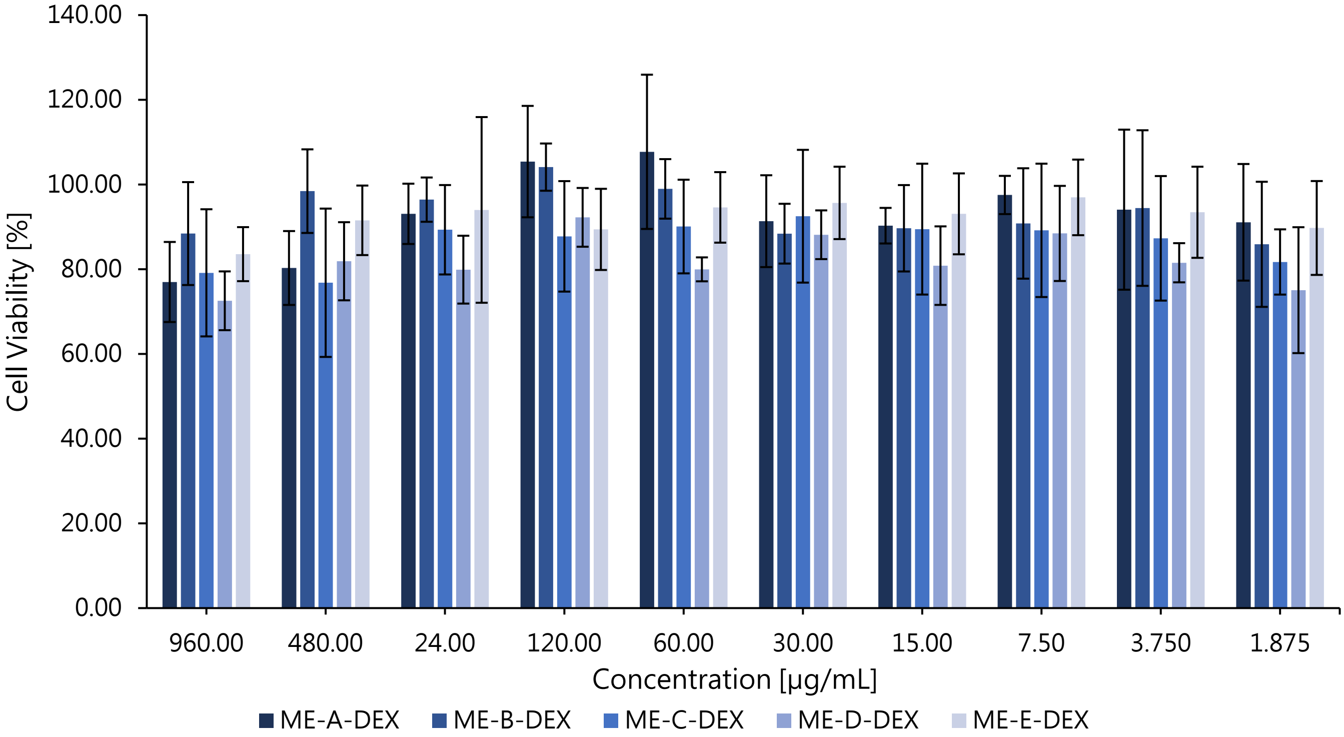

Figure S3. Mean cell viability of dexamethasone loaded ME samples in MTT assay as percentage of untreated control cells. HaCaT cells were treated for 24 h with ME with concentrations ranging from 1.875 µg/mL to 960 µg/mL. Samples were prepared in quadruplicates and the test was accomplished thrice. Data are shown as mean ± SD.

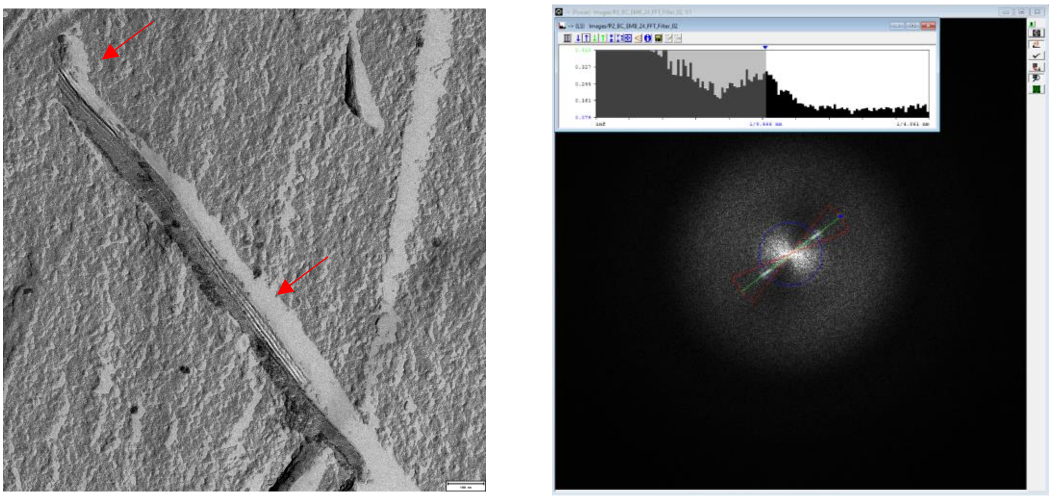

(**a**) (**b**)

Figure S4. (**a**) Fast fourier transform (FFT) filtered freeze-fracture transmission electron microscopy (FF-TEM) image of regular structures at the fracture surface due to layered assembly of cellulose protofibrils. (**b**) Measurement of repetition distance in FFT image.
